# Supplementary material for: Forecasting high-dimensional dynamics exploiting suboptimal embeddings
Source: Sci Rep. 2020 Jan 20;10:664. doi: 10.1038/s41598-019-57255-4 (PMC6971065; doi:10.1038/s41598-019-57255-4)
Supplement: Supplementary file 1 — Supplementary information. [file 41598_2019_57255_MOESM1_ESM.pdf]

Supplementary information for "Forecasting high-dimensional dynamics exploiting suboptimal embeddings"

Shunya Okuno[1,2,*], **Kazuyuki Aihara**[1,3,+], **and Yoshito Hirata**[3,4,+]

[1]Institute of Industrial Science, The University of Tokyo, 4-6-1 Komaba, Meguro-ku, Tokyo 153-8505, Japan
[2]Disaster Reduction & Environmental Engineering Department, Kozo Keikaku Engineering Inc., 4-5-3 Chuo, Nakanoku, Tokyo 164-0011, Japan
[3]International Research Center for Neurointelligence (WPI-IRCN), The University of Tokyo, 7-3-1 Hongo, Bunkyo-ku, Tokyo 113-0033, Japan
[4]Mathematics and Informatics Center, The University of Tokyo, 7-3-1 Hongo, Bunkyo-ku, Tokyo 113-0033, Japan
[*]okuno@sat.t.u-tokyo.ac.jp
[+]these authors contributed equally to this work

Effect of multiple optimizations

We solve multiple combinatorial optimization problems to divide the objective function into K problems in the first step. This procedure yields diverse embeddings to improve the performance of the combined forecast. Here, we numerically show the effect of the multiple optimizations.

We profiled the effect of K times optimization with an example of the 10-dimensional Lorenz'96I model with the data length of 4000. We forecasted the data by two methods: one is the proposed K optimizations, and the other is a single optimization to minimize the whole training in-sample error. All other processes follow the proposed forecast framework. Note that the computational time is almost equivalent with both methods.

As shown in Figures S1(a) and (b), the procedure with K optimizations enhanced the diversity of embeddings compared with the single optimization. This result suggests that the K times optimization found different but useful embeddings for each optimization process. The K times optimization also reduced the number of forecasts to combine (Figures S1(c) and (d)). This is because the single optimization process yielded similar embeddings, and the combination of similar forecasts yields a small performance improvement empirically[1,2]. Reductions in the number of combined forecasts and the computational time are important, especially for real-time applications.

Sensitivity to model parameters

We analyzed the sensitivity to parameters on multiple optimizations. First, we calibrated the sensitivity to the number of split datasets K with different data length in Figure S2. The result shows that a larger K is preferable for larger data length. Note that values of K do not have a significant impact for large data length.

Second, we calibrated the sensitivity to the threshold value of Hamming distance θ in Figure S3. The result shows that a larger θ tends to be slightly preferable for larger data length. The aim of setting θ is to prune similar forecasts and yield diverse embedding. For smaller data length, the solutions obtained by the optimization can be diverse because it is difficult to yield the global optimal embedding based on limited samples. For such conditions, we do not need to set a large θ. On the other hand, the solutions tend to converge to the global optimal solution for larger data length, and similar embeddings tend to be pooled based on the optimization. Therefore, we can set a larger θ for larger data length to prune similar forecasts.

Application to low-dimensional dynamics

We applied the proposed forecast framework to low-dimensional datasets—namely, the Lorenz'63 equations[4] (three variables), the Rössler equations[5] (three variables), and the six-dimensional Lorenz'96I equations (six variables). We set the data length to 4000 as the database and evaluated forecasts up to 10 steps ahead with 500 samples for all the cases. See the next section for the detailed conditions.

As shown in Figure S4, the proposed framework did not always achieve the best performance. There is little difference between the proposed framework and state-dependent weighting (SDW) for the Lorenz'63 dataset (Figure S4(a)), and SDW achieved the best performance for the Rössler dataset (Figure S4(b)). In contrast, when we increased the number of variables

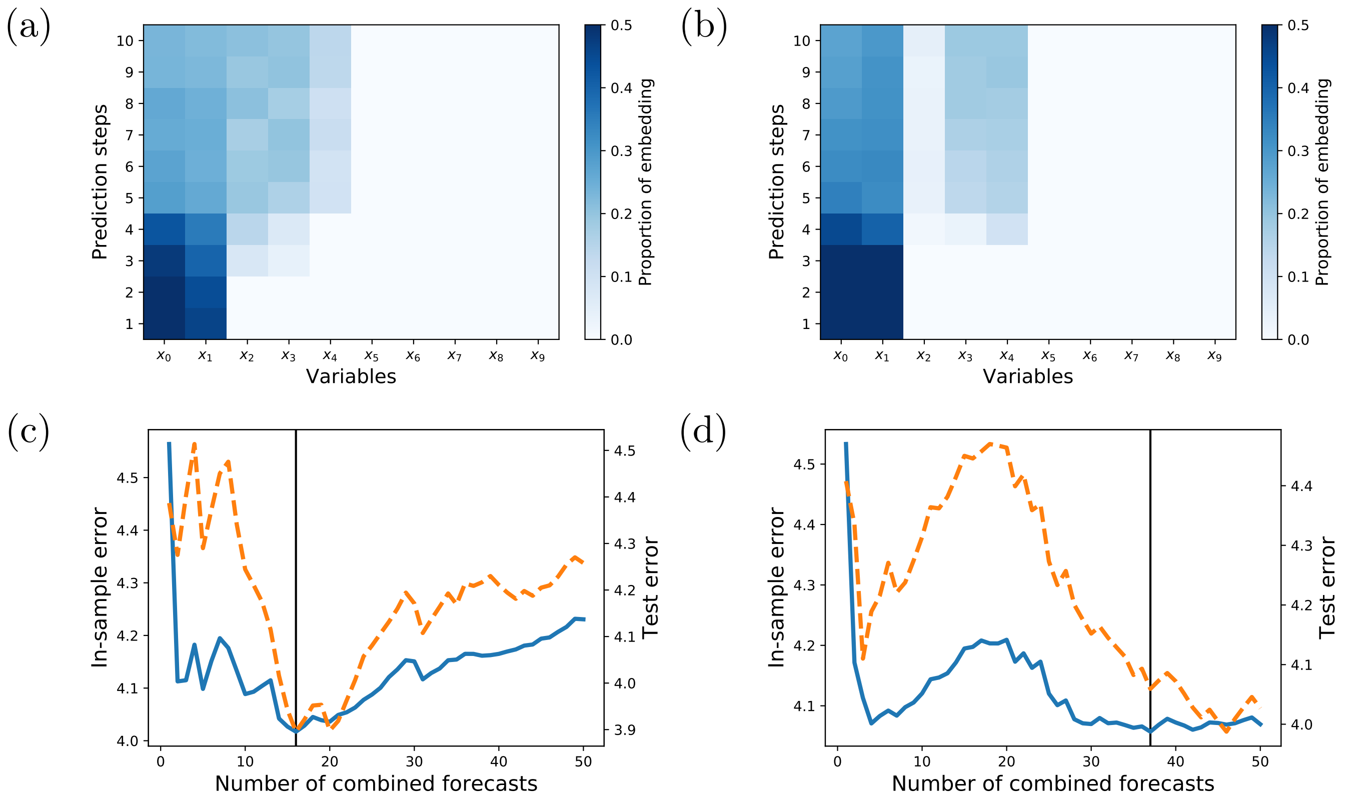

Figure S1. Effect of multiple optimizations: the proportion of embedding of the proposed forecasts with (a) K times optimization and (b) a single optimization to minimize the whole in-sample error. The color indicates the proportion of embedding for each variable averaged over the number of combined forecasts for each step. Panels (c) and (d) show the relation between the number of combined forecasts and the five-steps-ahead errors for (c) the K times optimization and (d) the single optimization, respectively. The solid line shows the in-sample error, and the dashed line shows the test error. The vertical solid line shows the selected number of combined forecasts.

to six, the proposed framework yielded the best performance (Figure S4(c)), and as stated in the main text, the proposed framework achieved much better results than the others for cases with 10 or 20 variables.

Interestingly, the performance of SDW was superior to that of MVE for all of the low-dimensional toy models mentioned above, although the performance of SDW for the high-dimensional dataset was not satisfactory, as stated in the main text. This result means that weighting based on the forecast performance and its corresponding state[6] worked fine with simple low-dimensional data. On the other hand, it is difficult to improve the performance by weighting for high-dimensional data, and a simple average is sufficient for such cases. In short, it is preferable to apply the proposed framework to complex high-dimensional and less noisy data, but it is worth considering the application of weighting methods such as SDW to simple low-dimensional data.

Diversity of embeddings for split dataset

We profiled the diversity of embeddings across the split dataset. We first analyzed the result of the 10-dimensional Lorenz'96I with data lengths of 1000 and 4000. We tested two parameter values for each case, namely the number of data split $K = 5$ and $K = 10$, and selected 30 suboptimal embeddings for all cases. We computed the Hamming distance matrix for the suboptimal embeddings to treat each embedding as a one-dimensional binary series. As shown in Figure S5, there are almost no repeated embeddings across segments for all cases. Although similar embeddings can be obtained as the data length increases, only one duplicate embedding exists in total. This suggests the difficulty of obtaining the optimal solution in such huge solution space for high-dimensional dynamics.

We also evaluated the result of the Lorenz'63 with data lengths of 1000 and 4000 in Figure S6. In these cases, the overall Hamming distance is smaller than that of the Lorenz'96I, and several overlapping embeddings are obtained. As the data length increases, the number of the overlapping embeddings increases. For such low-dimensional cases, the proposed framework is competitive with conventional methods, and we do not need to apply the proposed framework. This result is consistent with

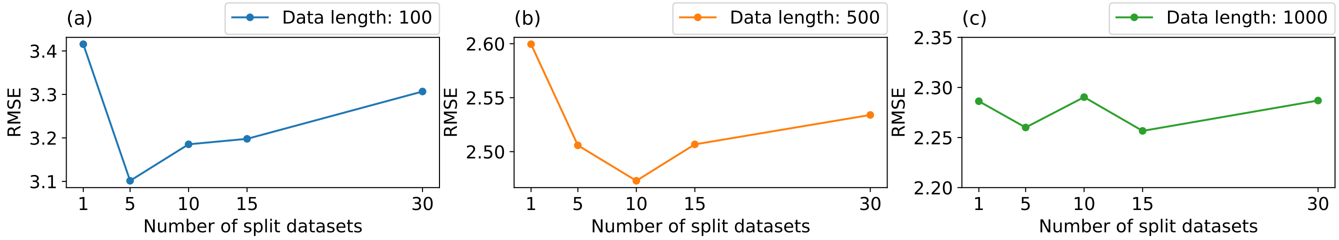

Figure S2. Sensitivity to the number of split datasets K on 10-dimensional Lorenz'96I equations[3] with data lengths of (a) 100, (b) 500, and (c) 1000. These tests were carried out using 20 datasets generated with different random initial conditions and evaluated by the median of the five-steps-ahead forecast error.

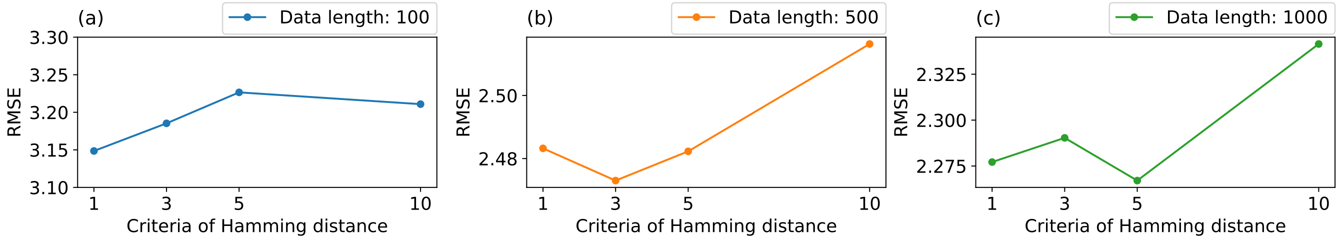

Figure S3. Sensitivity to the threshold value of Hamming distance θ on the 10-dimensional Lorenz'96I equations with data lengths of (a) 100, (b) 500, and (c) 1000. These tests were carried out using 20 datasets generated with different random initial conditions and evaluated by the median of the five-steps-ahead forecast error.

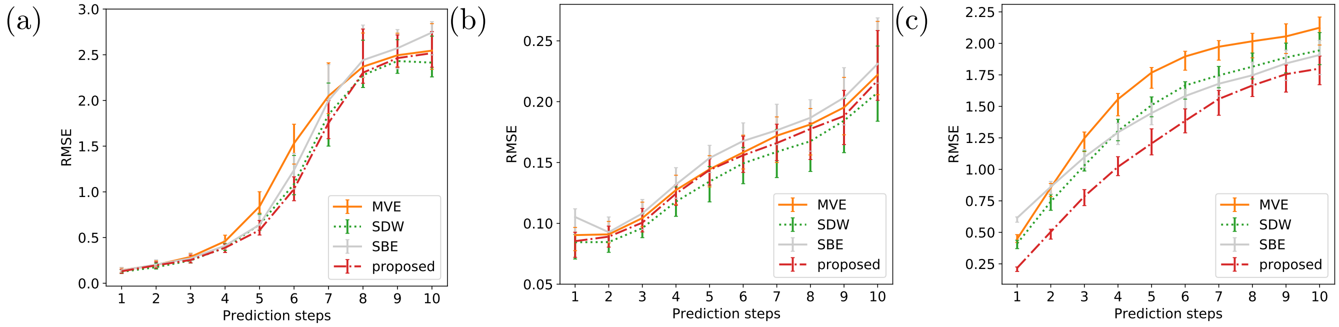

Figure S4. Forecast performance for low-dimensional datasets: RMSEs for (a) the Lorenz'63 dataset, (b) the Rössler dataset, and (c) the six-dimensional Lorenz'96I dataset. We compared the performance of multiview embedding (MVE), state-dependent weighting (SDW), and single-best embedding based on the $(\mu + \lambda)$-ES algorithm (SBE). These tests were carried out with 20 datasets generated with different random initial conditions and noise. The median, upper quartile, and lower quartile are shown.

that of the previous section.

Detailed conditions of the numerical experiments

Lorenz'96I equations

The Lorenz'96I equations[3] are expressed as 10-dimensional differential equations as follows:

$$\frac{dx_i}{dt} = x_{i-1}(x_{i+1} - x_{i-2}) - x_i + F, i = 0, 1, 2, ..., 9, \quad (1)$$

where F is a forcing variable and i is cyclic. We generated 10-dimensional time series: Lorenz'96I's x_0, x_1, x_2, x_3, and x_4 and random walks x_5, x_6, x_7, x_8, and x_9. We chose the initial condition from a normally distributed random value for each variable with $F = 8$, which is a common value to produce chaotic behavior. Details on this behavior are presented in Refs. 3, 7. We set the integration step to 0.001 and recorded every 50 points. Note that the initial transients were disregarded. We forecasted x_0

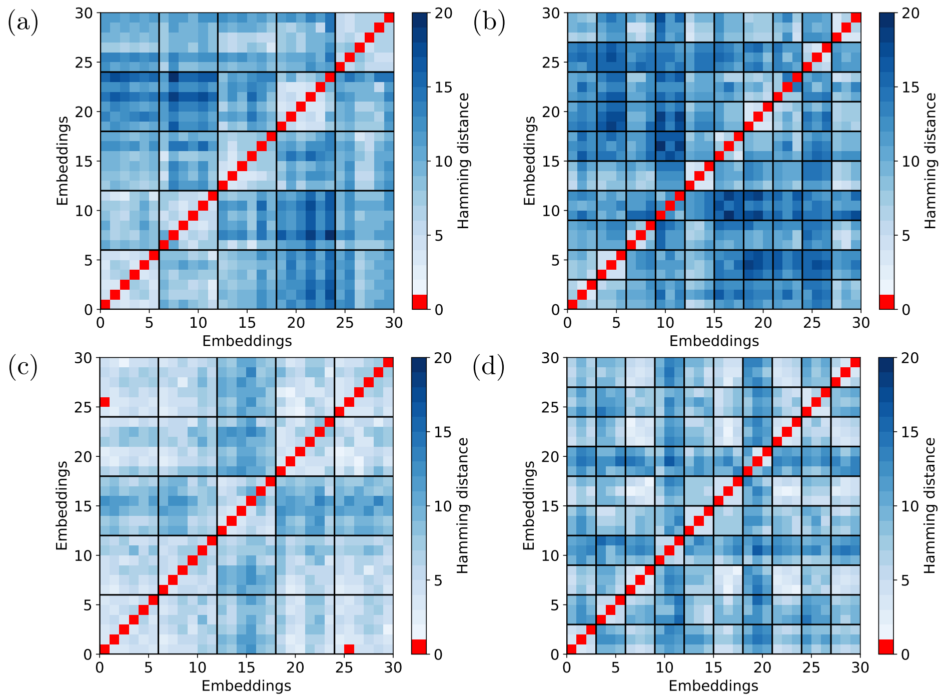

Figure S5. Hamming distance matrix of suboptimal embeddings on the 10-dimensional Lorenz'96I equations: (a) $K = 5$ with data length 1000, (b) $K = 10$ with data length 1000, (c) $K = 5$ with data length 4000, (d) $K = 10$ with data length 4000. We selected 30 embeddings in total for all cases. The Hamming distance was computed using a one-dimensional binary series. The red cells represent the same embeddings. The black lines depict the lines of data split.

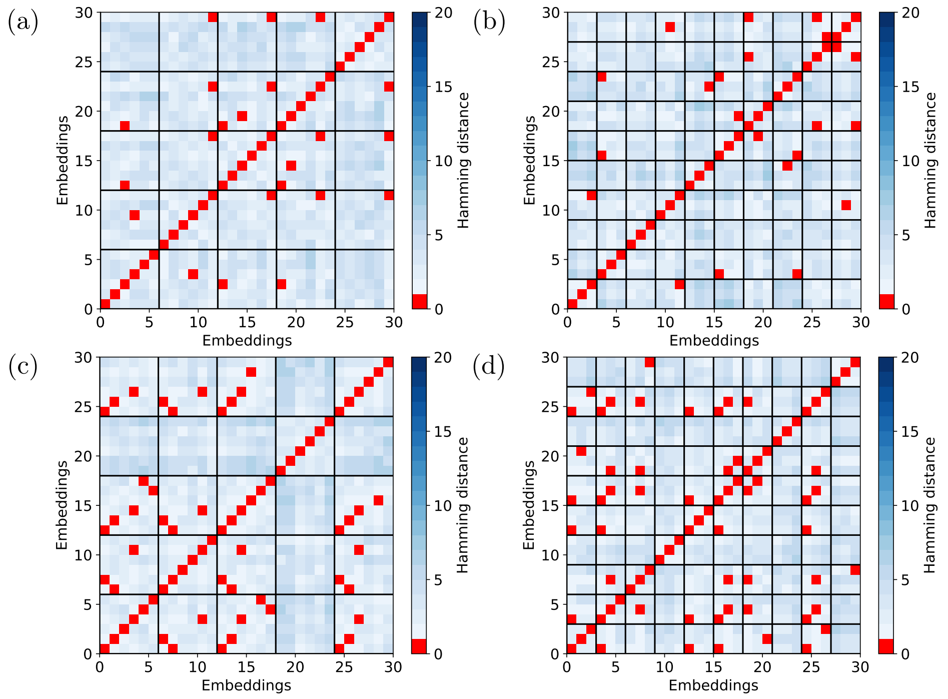

Figure S6. Hamming distance matrix of suboptimal embeddings on the Lorenz'63 equations: (a) $K = 5$ with data length 1000, (b) $K = 10$ with data length 1000, (c) $K = 5$ with data length 4000, (d) $K = 10$ with data length 4000. We selected 30 embeddings in total for all cases. The Hamming distance was computed by using a one-dimensional binary series. The red cells represent the same embeddings. The black lines depict the lines of data split.

up to 10 steps. We set $K = 10, M = 3, \theta = 3$, and $h_i(0) = 1, h_i(1) = \rho\ \forall i$ for $\rho \in \{0.0, -0.2, -0.4, -0.6, -0.8, -1.0\}$. For the $(\mu+\lambda)$-ES algorithm, we set $\mu = 50,\ \lambda = 100$, the number of generations to 10, and the number of populations to 100.

We compared the proposed framework with existing frameworks—namely, randomly distributed embedding with an aggregation scheme (RDE), multiview embedding (MVE), SDW with $E = 4$, and up to four lags for MVE and SDW. Because the possible number of embeddings combinatorially increases, we randomly generated 1000 embeddings instead of using brute-force calculation. Note that the total number of embedding evaluations is almost the same as that for the proposed framework.

We also computed three other methods to compare the performance of the proposed framework: (1) a conventional single variable embedding for the target variable with $E = 4$; (2) a multi-variable embedding to embed all variables with four lags; and (3) the single-best embedding via the $(\mu+\lambda)$-ES algorithm to minimize the total in-sample error within the whole training dataset. Note that these methods do not combine multiple forecasts.

Kuramoto–Sivashinsky equations

The Kuramoto–Sivashinsky equations[8,9] are expressed as follows:

$$\frac{\partial y}{\partial t} = -\frac{\partial^2 y}{\partial x^2} - \frac{\partial^4 y}{\partial x^4} - u\frac{\partial y}{\partial x}. \tag{2}$$

We computed $y(x,t)$ with spatially periodic boundary conditions in the interval $[0, L]$, where $L = 22$ and the number of uniform grids is 128. We generated 20-dimensional time series: the values of the first 10 grids of the Kuramoto–Sivashinsky equations $x_0, x_1, ..., x_9$ with a sampling time of 1.0 and 10 random walks $x_{10}, x_{11}, ..., x_{19}$. We set $E = 5$ and considered up to five lags for the existing schemes (RDE, MVE, and SDW). We forecasted x_0 up to 10 steps using the same parameter values, data length, and noise scales as those in the Lorenz'96I example.

Flood dataset

The flood forecasting competition dataset "Artificial Neural Network Experiment (ANNEX 2005/2006)"[10] contains river stage and rainfall data for three periods: 1993-10-01 to 1994-03-31, 1994-10-01 to 1995-03-31, and 1995-10-01 to 1996-03-31. We forecasted river stage Q 6, 12, 18, and 24 h ahead using nine variables: the river stages of the target (Q) and three upstream sites ($US1$, $US2$, and $US3$) and five rain gauges ($RG1$, $RG2$, $RG3$, $RG4$, and $RG5$). All data were sampled by 6 h. We used the period of 1994-10-01 to 1995-03-31 for testing and the others for training. We set $K = 6, M = 3, \theta = 3$, and $h_i(0) = 1, h_i(1) = \rho\ \forall i$ for $\rho \in \{0.0, -1.0\}$. For the $(\mu+\lambda)$-ES algorithm, we set $\mu = 50, \lambda = 100$, the number of generations to 20, and the number of populations to 100.

Lorenz'63 equations

The Lorenz'63 equations[4] are expressed as three-dimensional differential equations as follows:

$$\frac{dx}{dt} = p(y-x), \frac{dy}{dt} = x(r-z) - y, \frac{dz}{dt} = xy - bz. \tag{3}$$

We generated three-dimensional time series: Lorenz'63's x and y and a random-walk series. We set the integration step to 0.001 and recorded every 100 points with $p = 10, b = 8/3$, and $r = 28$. Note that the initial transients were disregarded. We forecasted x up to 10 steps. We set $K = 10, M = 3, \theta = 3$, and $h_i(0) = 1, h_i(1) = \rho\ \forall i$ for $\rho \in \{0.0, -0.2, -0.4, -0.6, -0.8, -1.0\}$. For the $(\mu+\lambda)$-ES algorithm, we set $\mu = 50, \lambda = 100$, the number of generations to 10, and the number of populations to 100.

We compared the proposed framework with MVE and SDW with $E = 4$ up to five lags and the single-best embedding via the $(\mu+\lambda)$-ES algorithm to minimize the total in-sample error within the whole training dataset. Note that we did not carry out calculations with RDE because we were not able to prepare a sufficient number of "nondelay embeddings" for this type of low-dimensional data.

Rössler equations

The Rössler equations[5] are expressed as three-dimensional differential equations as follows:

$$\frac{dx}{dt} = -y - z, \frac{dy}{dt} = x + ay, \frac{dz}{dt} = b + z(x-c). \tag{4}$$

We generated three-dimensional time series: Rössler x and y and a random-walk series. We set the integration step to 0.001 and recorded every 500 points with $a = 0.36, b = 0.4$, and $c = 4.5$. Note that the initial transients were disregarded. We forecasted x up to 10 steps with the same conditions as those of the Lorenz'63 equations.

Six-dimensional Lorenz'96I equations

The six-dimensional Lorenz'96I dataset contains the six-dimensional Lorenz'96I series x_0, x_1, and x_2 and random walks x_3, x_4, and x_5. The conditions for numerical integration were the same as those of the 10-dimensional Lorenz'96I equations. We forecasted x_0 up to 10 steps with the same conditions as those of the Lorenz'63 equations.

Method of analogues

We applied a variation of the method of analogues[11] to obtain a p-steps-ahead forecast $\hat{y}_f(t+p|t)$ at time t. The method of analogues takes the forward paths of neighboring trajectories as the forecast. Here, we forecast $y_f(t+p|t)$ from the set of delay coordinates $\{v(t) \mid t \in \mathscr{T}_{train}\}$. We first search the database to find the neighboring points of $v(t)$. Then, the method of analogues gives $\hat{v}(t+p|t)$ in terms of the set of neighboring time indices $\mathscr{I}(t)$ as follows:

$$\hat{v}(t+p|t) = \sum_{t' \in \mathscr{I}(t)} \lambda(t') v(t'+p), \tag{5}$$

where $\lambda(t') \in \mathbb{R}_+$ is a weight satisfying $\sum_{t' \in \mathscr{I}(t)} \lambda(t') = 1$. We employ the following weight for a neighboring time index T throughout this paper.

$$\lambda(T) = \|v(T) - v(t)\|_2^2 / \sum_{t' \in \mathscr{I}(t)} \|v(t') - v(t)\|_2^2. \tag{6}$$

Note that we can apply more advanced methods such as those in Refs. 12, 13 or any other regression methods with our proposed forecasting framework.

References

1. Sollich, P. & Krogh, A. Learning with ensembles: how over-fitting can be useful. In *Advances in neural information processing systems*, 190–196 (1996).
2. Kuncheva, L. I. & Whitaker, C. J. Measures of Diversity in Classifier Ensembles and Their Relationship with the Ensemble Accuracy. *Mach. Learn.* **51**, 181–207, DOI: 10.1023/A:1022859003006 (2003).
3. Lorenz, E. N. Predictability: a problem partly solved. In *Seminar on Predictability*, 1–18 (ECMWF, Reading, England, 1996).
4. Lorenz, E. N. Deterministic nonperiodic flow. *J. Atmospheric Sci.* **20**, 130–141 (1963).
5. Rössler, O. E. An equation for continuous chaos. *Phys. Lett. A* **57**, 397–398 (1976).
6. Okuno, S., Aihara, K. & Hirata, Y. Combining multiple forecasts for multivariate time series via state-dependent weighting. *Chaos: An Interdiscip. J. Nonlinear Sci.* **29**, 33128, DOI: 10.1063/1.5057379 (2019).
7. Karimi, A. & Paul, M. R. Extensive chaos in the Lorenz-96 model. *Chaos: An Interdiscip. J. Nonlinear Sci.* **20**, 43105, DOI: 10.1063/1.3496397 (2010).
8. Kuramoto, Y. & Tsuzuki, T. Persistent Propagation of Concentration Waves in Dissipative Media Far from Thermal Equilibrium. *Prog. Theor. Phys.* **55**, 356–369, DOI: 10.1143/PTP.55.356 (1976).
9. Sivashinsky, G. I. Nonlinear analysis of hydrodynamic instability in laminar flames-I. Derivation of basic equations. *Acta Astronaut.* **4**, 1177–1206, DOI: 10.1016/0094-5765(77)90096-0 (1977).
10. Dawson, C. *et al.* A comparative study of artificial neural network techniques for river stage forecasting. In *Proceedings of the International Joint Conference on Neural Networks*, vol. 4, 2666–2670, DOI: 10.1109/IJCNN.2005.1556324 (IEEE, Montreal, Canada, 2005).
11. Lorenz, E. N. Atmospheric predictability as revealed by naturally occurring analogues. *J. Atmospheric Sci.* **26**, 636–646 (1969).
12. Farmer, J. D. & Sidorowich, J. J. Predicting chaotic time series. *Phys. Rev. Lett.* **59**, 845–848, DOI: 10.1103/PhysRevLett.59.845 (1987).
13. Hirata, Y. *et al.* Approximating high-dimensional dynamics by barycentric coordinates with linear programming. *Chaos* **25**, 013114, DOI: 10.1063/1.4906746 (2014).
